# Machine Learning for Property Prediction and Optimization of Polymeric Nanocomposites: A State-of-the-Art

**DOI:** 10.3390/ijms231810712

**Published:** 2022-09-14

**Authors:** Elizabeth Champa-Bujaico, Pilar García-Díaz, Ana M. Díez-Pascual

**Affiliations:** 1 Universidad de Alcalá, Departamento de Teoría de la Señal y Comunicaciones, Ctra. Madrid-Barcelona Km. 33.6, 28805 Alcalá de Henares, Madrid, Spain; 2 Universidad de Alcalá, Facultad de Ciencias, Departamento de Química Analítica, Química Física e Ingeniería Química, Ctra. Madrid-Barcelona Km. 33.6, 28805 Alcalá de Henares, Madrid, Spain

**Keywords:** machine learning, artificial neural network, carbon nanomaterials, polymer nanocomposites, property prediction, optimization

## Abstract

Recently, the field of polymer nanocomposites has been an area of high scientific and industrial attention due to noteworthy improvements attained in these materials, arising from the synergetic combination of properties of a polymeric matrix and an organic or inorganic nanomaterial. The enhanced performance of those materials typically involves superior mechanical strength, toughness and stiffness, electrical and thermal conductivity, better flame retardancy and a higher barrier to moisture and gases. Nanocomposites can also display unique design possibilities, which provide exceptional advantages in developing multifunctional materials with desired properties for specific applications. On the other hand, machine learning (ML) has been recognized as a powerful predictive tool for data-driven multi-physical modelling, leading to unprecedented insights and an exploration of the system’s properties beyond the capability of traditional computational and experimental analyses. This article aims to provide a brief overview of the most important findings related to the application of ML for the rational design of polymeric nanocomposites. Prediction, optimization, feature identification and uncertainty quantification are presented along with different ML algorithms used in the field of polymeric nanocomposites for property prediction, and selected examples are discussed. Finally, conclusions and future perspectives are highlighted.

## 1. Introduction

**Figure 1:**
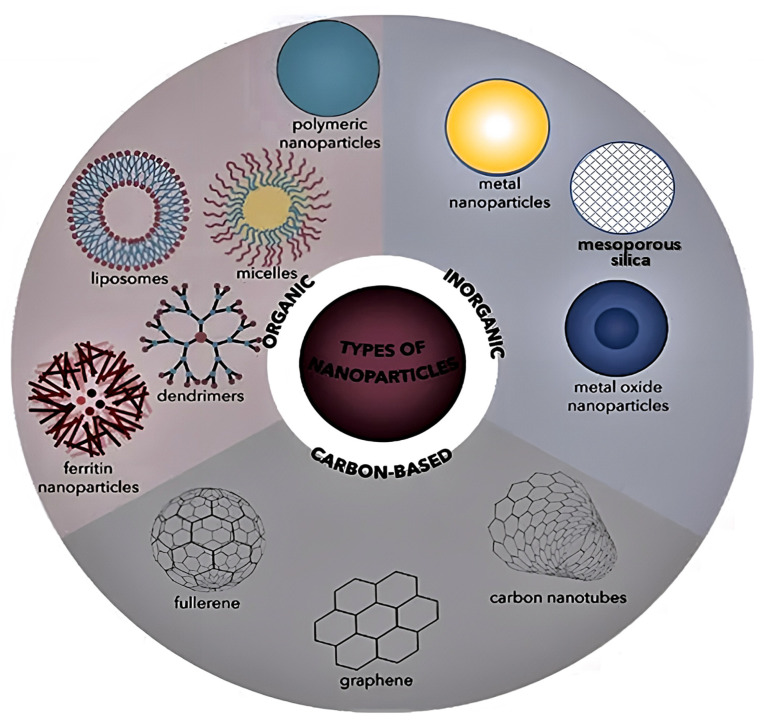
Classification of the main types of nanomaterials according to their nature into organic, inorganic and carbon-based.

The field of nanocomposite materials is currently an area of strong activity that promises to have far-reaching impacts on our society. Amongst the extraordinary range of developing research lines, the introduction of nanofillers into polymers in order to impart specific and noticeable property improvements is still demonstrating important advances [1,2]. These nanocomposite materials exhibit significant enhancements in mechanical, electrical and thermal properties compared to composite materials incorporating conventional fillers, such as glass, carbon or aramide fibres, and are currently applied in automobile, aeronautical, aerospace, marine, civil and many other technological applications [3,4,5] that request an outstanding combination of mechanical and thermal properties. Polymer nanocomposites are made up of two phases: the matrix phase (continuous) and the nanoreinforcement phase (dispersed), with sizes in the range of 1–100 nm. Usually, a thermosetting or thermoplastic polymer acts as the matrix with the aim to transfer the load uniformly to the embedded nanoreinforcement [6]. Different types of nanomaterials are used to strengthen the polymeric matrix and are known as nanofillers or nanoreinforcing agents. According to their nature, these nanofillers can be classified into three main groups, as depicted in Figure 1: (1) organic, including dendrimers, micelles, liposomes, polymer nanoparticles (NPs) and ferritin; (2) inorganic, including metal NPs (Ag, Au, Cu), metal oxide NPs (e.g., Fe_3_O_4_, ZnO, MgO, TiO_2_) and mesoporous silica; (3) and carbon-based, including fullerenes, quantum dots, carbon nanotubes, graphene and its derivatives [7,8].

According to their dimensions [9], nanomaterials can be classified as 0D when all their dimensions are smaller than 100 nm, such as fullerenes, quantum dots (QDs) and metallic NPs, 1D when there are two dimensions smaller than 100 nm, such as carbon nanotubes (CNTs), 2D when only one dimension is on the nanoscale, such as graphene, and 3D when they are not confined to the nano-scale in any dimension, such as dendrimers.

**Figure 2:**
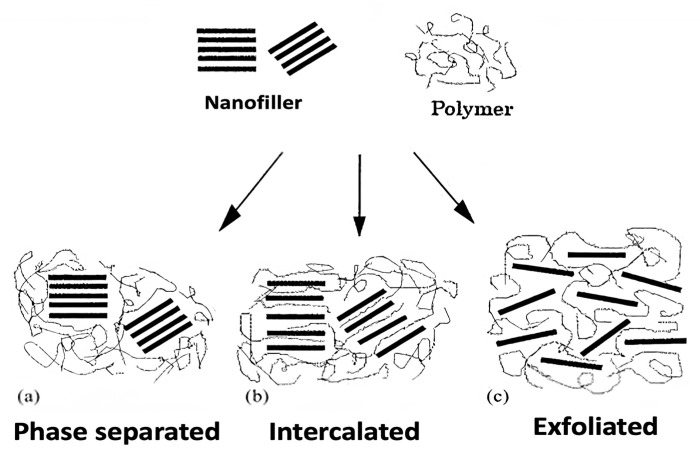
Possible structures of polymer nanocomposites: (**a**) phase separated, (**b**) intercalated and (**c**) exfoliated.

The ideal design of a nanocomposite involves individual nanoparticles homogeneously dispersed in a polymer matrix. The dispersion state of nanoparticles is the key challenge to attaining the full potential of property enhancement [10,11]. A uniform nanofiller dispersion would lead to a large interfacial area (interface) between the nanomaterial and the chains of the neat polymer, which is expected to result in improved properties compared to conventional polymer composites incorporating macro- or micro-fillers. The reinforcing effect of the nanofiller is attributed to several factors, such as nature and type of nanofiller, the concentration of nanofiller and polymer, nanofiller aspect ratio, geometry, size, orientation and distribution, etc. [12,13]. The assessment of the nanofiller dispersion in the polymer matrix is crucial, given that the mechanical and thermal properties are strongly related to the morphologies obtained. In this regard, three types of nanocomposite morphologies have been observed (Figure 2) [14]: phase separated, intercalated and exfoliated nanocomposites. When the polymer is unable to intercalate between the nanofiller, a composite of separate phases is attained (Figure 2a), with comparable properties to those observed in traditional composites. An intercalated structure, in which a single extended polymer chain is intercalated between the nanofiller, results in a well-ordered intercalated morphology (Figure 2b). When the nanofiller is completely and uniformly dispersed in a continuous polymer matrix, an exfoliated structure is obtained (Figure 2c). An important aspect of these nanocomposites is that property improvements are attained at very low reinforcement loadings (typically 1–10 wt.%) [15].

**Figure 3:**
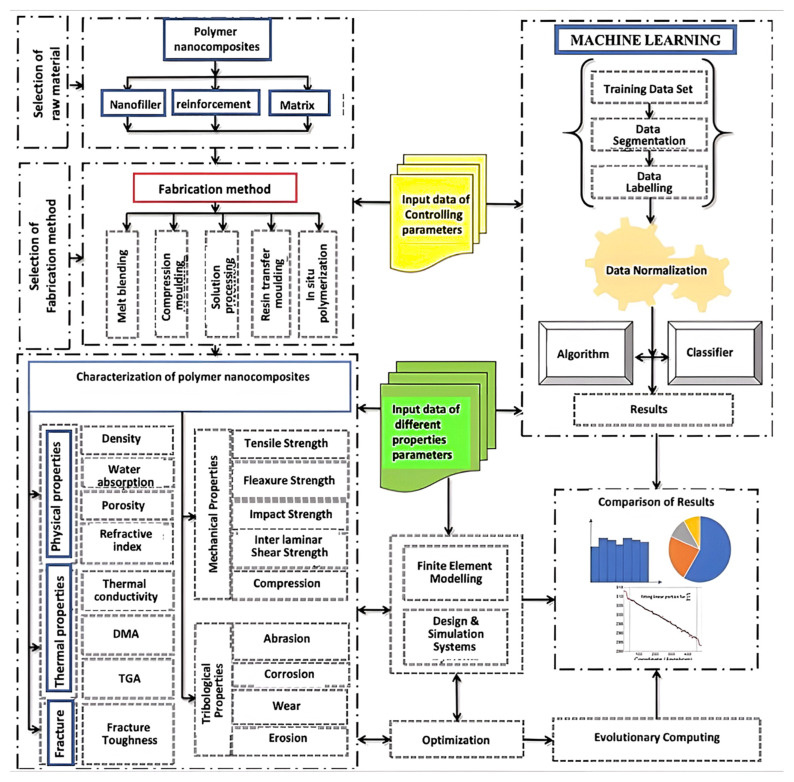
Application of machine learning for predicting the properties of polymeric nanocomposites.

The manufacturing process of the nanocomposites is also a key factor conditioning the mechanical response together with the long-term performance of the resulting nanocomposites. Melt-blending, compression moulding, solution processing, resin transfer moulding and in-situ polymerization are the processes commonly used to prepare polymeric nanocomposites (Figure 3) [16]. The choice of manufacturing process depends on the intended application of the final product. Another parameter determining the mechanical behaviour of nanocomposite materials is the residual stress and strain [17]. Stress transfer from the continuous phase to the dispersed phase is a very important phenomenon that critically affects the strength and stiffness of the composites, and is determined by their difference in the elastic modulus and the Poisson’s ratio [18]. The coefficient of the thermal expansion of the matrix and the reinforcement is also needed to be taken into account since a mismatch in this coefficient may lead to the development of thermal residual stresses [19].

Polymer nanocomposites have synergistic properties that can easily be tailored for attaining a desirable specific set of properties by selecting the appropriate combination of continuous and dispersed phases. For optimization and material design, all the processing parameters should be taken into account simultaneously. Modelling the complex relationships between the governing parameters (both input and output) is very arduous. Despite the availability of large experimental setups and computational tools, it is tedious and time-consuming to explore the significance of each of the governing parameters experimentally. Over the last two decades, material science has experienced a progressive shift from developing raw computational techniques for the design of novel materials to developing coupled methods that improve the results’ reliability via computational predictions and experimental validation. Finite element and molecular dynamics simulations have been applied to model the material behaviour in numerous arenas; however, the complexity and computational intensiveness of the approaches have prompted researchers to look for additional alternatives [20,21,22]. Therefore, many scholars have relied on the machine learning approach to determine the implication of the process parameters for an optimal design [23]. Machine learning (ML) is a subset of artificial intelligence that provides systems with the ability to automatically learn and improve from experience without being explicitly programmed. It is trained on huge amounts of data and sets linkages between input fingerprints and output properties, thus offering a powerful surrogate model for structure–property analysis [24,25,26,27,28]. ML offers a wider scope for effectively analysing the behaviour of resulting composites with limited experimentation or computationally intensive realizations of expensive models (Figure 3).

The application of ML to polymeric nanocomposites enables us to predict numerous multifunctional properties based on both the components and their proportions. Many ML algorithms have been developed for polymer composites depending on the property types and the datasets available. However, most of the studies are restricted in scope by the constraints of multiple variables which result in increased dimensionality and uncertainty caused by the randomness of the data.

This paper aims to provide a brief overview of the most important findings related to the application of ML for the rational design of polymeric nanocomposites. First, different types of nanomaterials used in polymer nanocomposites are described. Then, prediction, optimization, feature identification and uncertainty quantification are presented along with different ML algorithms used in the field of polymeric nanocomposites for property prediction, and selected examples are discussed. Finally, conclusions and future perspectives are highlighted.

## 2. Nanofillers in Polymeric Nanocomposites: Properties and Synthesis Methods

### 2.1. Carbon-Based Nanofillers

**Figure 4:**
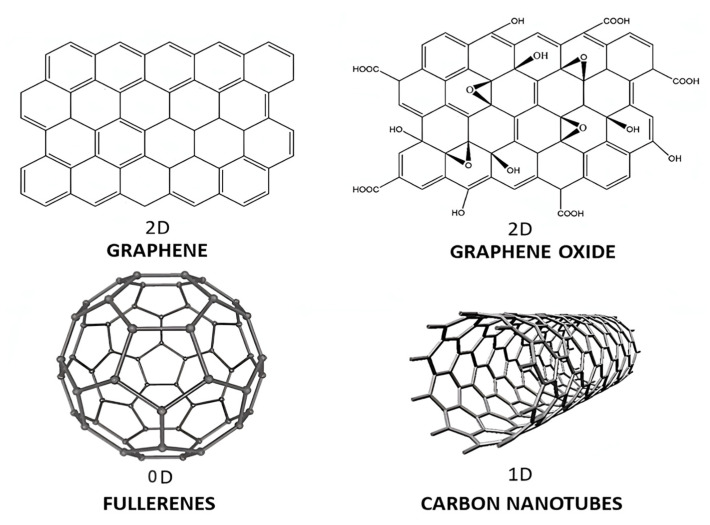
Representation of the structure of carbon-based nanomaterials: 2D graphene (G) and graphene oxide (GO), 0D fullerenes and 1D carbon nanotubes (CNTs).

Different allotropes from carbon have been recently used as nanofillers in polymeric nanocomposites, including fullerenes, quantum dots (QDs), carbon nanotubes (CNTs), graphene (G) and its derivatives graphene oxide (GO) and reduced graphene oxide (rGO) (Figure 4). The synthesis method of carbon-based nanomaterials strongly influences their purity and quality, hence the final composite properties [9,29,30].

#### 2.1.1. Fullerenes

Fullerenes were reported for the first time at Sussex University by Kroto and Smalley in 1985, when they were working with the sooty residue produced by vaporising carbon in a helium atmosphere [31]. They contain fused rings of five to seven atoms, are spherical or ellipsoid in shape with a hollow structure and have sp^2^ and sp^3^ carbon atoms. The mass spectrum of the residue showed peaks corresponding to ball-like polyhedral molecules which they called “buckyballs”. The most known is C_60_, named Buckminster fullerene [32]. It consists of a truncated icosahedron, bearing a resemblance to a football ball made of twenty hexagons and twelve pentagons.

Their structure is characteristic since it is borderless, uncharged, and lacks of boundaries or unpaired electrons. These characteristics distinguish fullerenes from other carbon allotropes, such as graphite or diamond, which have electrical charges and edges with dangling bonds. Fullerenes are soluble in common organic solvents, such as toluene, chlorobenzene and 1,2,3-trichloropropane at room temperature [33]. They are chemically reactive and can be combined with polymers to form nanocomposites with new thermal and mechanical properties.

#### 2.1.2. Quantum Dots

Quantum dots (QDs) are 0D semiconductor nanoparticles with optical and electronic properties that differ from those of larger particles due to a quantum effect in which the electrons are confined in all directions [34]. When QDs are irradiated with UV light, an electron is excited from the valence to the conduction band, and when it goes back to the ground state, it emits electromagnetic radiation; this process is known as “photoluminescence”.

Carbon QDs were accidentally discovered in 2004 by Xu et al. [35] during the purification of single-walled carbon nanotubes. Besides their fluorescent characteristics, they have high stability, good conductivity, good biocompatibility and environmental friendliness, and have been extensively investigated for applications in many fields, including diode lasers, solar cells, LEDs, inkjet printings, electron transistors, amplifiers and biological sensors, microscopy and medical imaging [36]. They can be used as donor fluorophores in Föster resonance energy transfer. Moreover, their improved photostability allows for the development of highly sensitive devices for cellular imaging, enabling the acquisition of high-resolution 3D images. They can be employed for tumour targeting under in vivo conditions [24], biomedicine, optronics, catalysis, and sensing [37].

**Figure 5:**
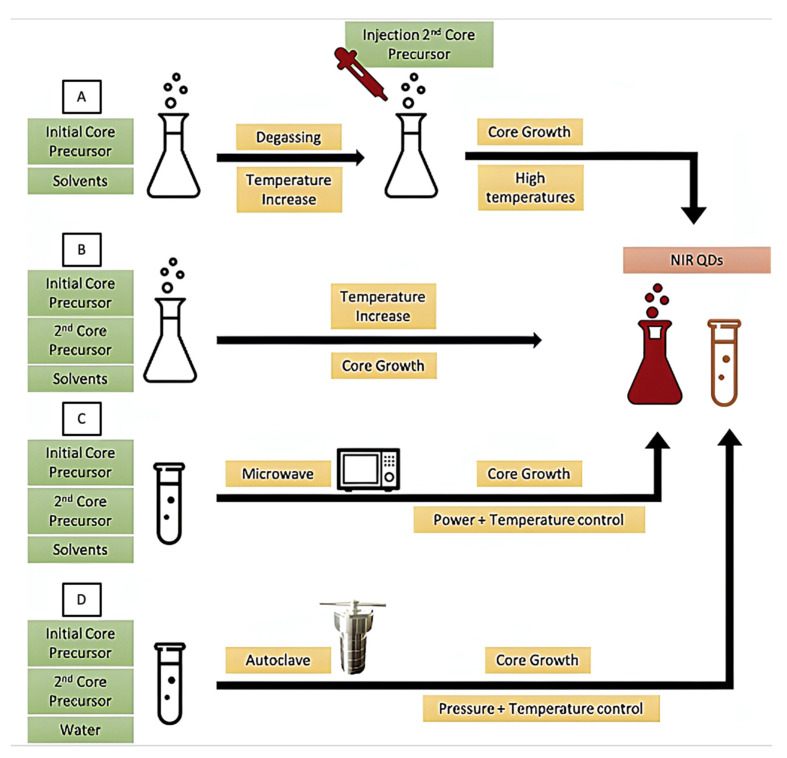
Methods used for QD synthesis. (A) hot-inject method; (B) heat-up approach; (C) microwave method; (D) hydrothermal method. Reprinted from Ref. [38], copyright 2021, with permission from Elsevier.

The main methods used for CQD preparation are hot-injection, heat-up, microwave and hydrothermal synthesis (Figure 5) [38]. The hot-injection method (Figure 5A) was first introduced by the works of Murray and co-workers [34], and is the most used to synthesize a wide variety of monodisperse QDs. However, this method has some shortcomings: the high temperature of the reaction results in fast reaction rates, hence the mixing of the reagents must be efficient to produce monodisperse nanoparticles. Moreover, this method is not suitable for large-scale QD production.

The heat-up approach (Figure 5B) is a single-pot reaction without an injection step. This results in high polydispersity in size distribution. Another drawback is the need to employ reagents that have similar reactivities at the desired reaction temperatures. The microwave method (Figure 5C) uses electromagnetic radiation to achieve a rapid and homogeneous heating of the reaction. The control over the heating rate enables one to attain monodisperse QDs [39]. The hydrothermal method (Figure 5D) uses aqueous solvents at a high temperature and pressure, which leads to the rapid formation of nuclei, resulting in monodisperse QDs.

#### 2.1.3. Carbon Nanotubes

CNTs were first reported by Iijima in 1991 [40]. They consist in 1D, rolled-up layers of carbon atoms with sp^2^ hybridization (Figure 4), and can be classified into single-walled carbon nanotubes (SWCNTs), one-piece cylinders with only one carbon layer, double-walled carbon nanotubes (DWCNTs), with two concentric carbon layers or multi-walled carbon nanotubes (MWCNTs), with several concentric carbon layers linked by weak interactions. They have a low density (1.3 g/cm^3^) and outstanding mechanical, thermal and electrical properties which depend on their diameter, length and chirality [41]. Their stiffness is the highest amongst any known material, with a Young’s modulus close to 1 TPa and strength of about 30 GPa [42]. Depending on their chirality, they can be conducting, semiconducting or insulating. The conducting ones have a current density in the order of 4 × 10^9^ A/cm^2^, much higher than that of metals such as Ag (10^5^ A/cm^2^). They also show a very high thermal conductivity (more than 10^3^-fold that of metals such as Cu), and display very high thermal stability, up to 700 °C under an air atmosphere and 2800 °C under a vacuum [43]. However, they have a great predisposition to aggregate and form ropes, which leads to properties worsening, particularly mechanical and electrical. Henceforth, functionalization with polymers [44] or other molecules is frequently required.

The most common methods to synthesize CNTs are chemical vapor deposition (CVD), electric arc discharge and laser ablation [45,46]. CVD is a technique in which the vaporized reactants (hydrocarbon gases) react chemically inside a quartz tube filled with inert gas, which is placed in a furnace kept at high temperature (500–900 °C). The hydrocarbon gases are pumped into the quartz tube, undergo a pyrolysis reaction and form vapor carbon atoms that deposit onto a substrate with metal catalyst nanoparticles of Fe, Co and Ni. The obtained CNTs are typically purified to obtain the raw CNTs.

In the arc discharge approach, a potential is applied across pure graphite electrodes maintained at a high pressure of inert gas filled inside a quartz chamber. When the electrodes strike each other, an electric arc is generated and the energy is transferred to the anode, which ionizes the carbon atoms of pure graphite and produces C^+^ ions in the form of plasma. These positively charged ions move towards the cathode, where are reduced, deposited and grown as CNTs [47].

The laser ablation method is a physical vapor deposition method in which a graphite target placed at a quartz chamber filled with inert gas is vaporized by a laser source. The vaporized target atoms are swept toward a cooled copper collector by the flow of the inert gas, where are they deposited and grow [47].

#### 2.1.4. Graphene and Its Derivatives

Graphene (G) is a 2D atomically thick carbon nanomaterial comprising a honeycomb lattice of sp^2^ carbon atoms [48]. It was discovered by Novoselov and Geim at Manchester University in 2004, while exfoliating a graphite pencil with Scotch tape [49]. G has outstanding electrical, optical and thermal properties, combined with a high mechanical resistance, transparency, low density and flexibility. For instance, it has a thermal conductivity in the range of 3000–5000 W m^−^^1^ K^−^^1^ [50], about 10-fold higher than that of other metals such as Cu, a very high electron mobility (20,000 cm^2^ V^−^^1^ s^−^^1^) and exceptional electrical conductivity (up to 5000 S cm^−^^1^). Moreover, it is one the strongest materials on earth, with a Young’s modulus of around 1 TPa and tensile strength of about 120 GPa, significantly stiffer than steel [51]. Additionally, it is a zero-gap semiconductor, electroactive and transparent, absorbing just 2% of the incident light.

These exceptional properties make G a perfect candidate for many applications, such as sensors, supercapacitors, fuel cells, photovoltaic devices, batteries, nanocomposites, flexible electronic devices and so forth [52,53].

G can be modified with polymers via covalent and non-covalent approaches to form functional nanocomposites. Covalent interactions happen via the formation of chemical bonds, through approaches named as “grafting-from” and “grafting-to” [4,5]. In the first, G is used as a growing point for the polymer chains, while in the second there is a direct coupling of G with the polymer chains, which should incorporate reactive functional groups. Nevertheless, these strategies can change the aromatic π-system of G and generate defects that result in a poorer performance. On the other hand, the non-covalent approach consists in the adsorption of polymers onto G via weak interactions, such as H-bonding, hydrophobic (van der Waals), H- π, cation- π and so forth.

**Figure 6:**
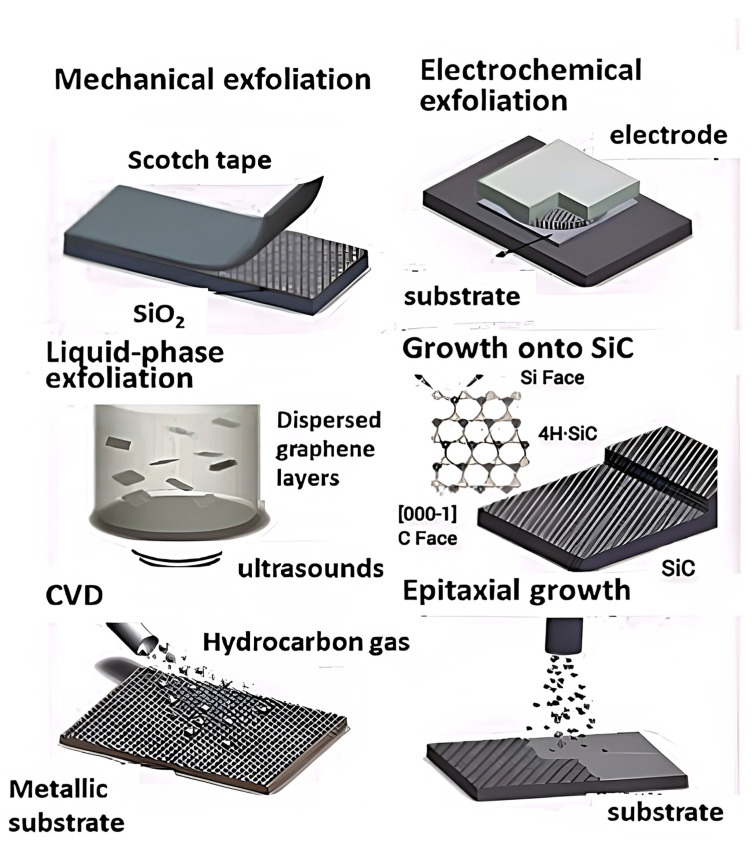
Top-down and bottom-up techniques for graphene synthesis.

G synthesis is typically performed by two ways [54], namely the “bottom-up” and “top-down” approaches (Figure 6). In the top-down methods, the initial material is graphite, which can be exfoliated mechanically (scotch tape method), in liquid phase (typically with the aid of ultrasounds to disperse the graphene layers [55,56]) or electrochemically, which is based on the penetration of graphite by ions from the electrochemical solution using a potential, as depicted in Figure 6 [57,58].

The bottom-up techniques rely on making graphene from molecular precursors by chemical vapor deposition (CVD) or epitaxial growth. CVD is an economic and large-scale method to yield high-quality graphene, even though it is hard to control the thickness of its films [59]. A hydrocarbon gas is saturated at very high temperatures on a substrate made of a transition metal. When it cools down, the solubility of carbon decreases, and the graphene film is made. Epitaxial growth is one of the most expensive methods since it requires a SiC substrate that is heated at very high temperature. However, it enables a precise control over the film thickness via tailoring the process parameters.

On the other hand, graphene derivatives are currently used for numerous applications, including the fabrication of biosensors. Amongst them, the most important is graphene oxide (GO), and the oxidized form of G with oxygenated functional groups, mainly carboxylic groups on the edges and epoxy and hydroxyl groups on the layer plane, typically synthesized via Hummer´s method using strong oxidizing agents, such as sulfuric or nitric acid [60,61]. Another well-known derivative is reduced graphene oxide (rGO), which is obtained via the thermal treatment of GO to remove functional groups [62] or by the chemical reduction of GO using synthetic reducing agents, such as hydrazine or sodium borohydride, or more recently eco-friendly, natural reducing agents, such as aminoacids (i.e., ascorbic acid) or plant extracts.

### 2.2. Inorganic Nanofillers

#### 2.2.1. Layered Nanoclays

**Figure 7:**
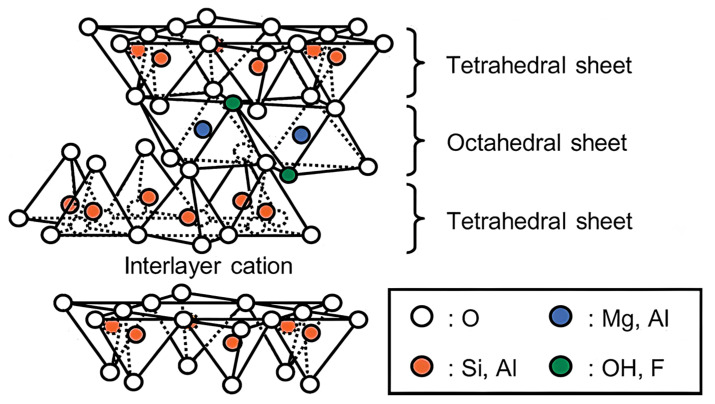
Structure of 2:1 layered silicates. Reprinted from Ref. [66], copyright 2014, with permission from Elsevier.

Nanoclays belong to a class of materials made of layered silicates or clay minerals with traces of metal oxides and organic matter. Clay minerals are hydrous aluminum phyllosilicates with adjustable amounts of iron, magnesium, alkali metals, alkaline earths and others cations [63]. Clays have been found to be effective reinforcing fillers for polymer due to their lamellar structure and high specific surface area (750 m^2^/g) [11]. Thus, over the past years, it has been reported that the dispersion of exfoliated clays in polymer leads to a remarkable increase in stiffness, fire retardancy and barrier properties at a very low nanoparticle volume fraction [64]. Examples of clays are montmorillonite, saponite, laponite, hectorite, sepiolite and vermiculite [65]. Among them, montmorillonite (MMT) is the most widely used in polymer nanocomposites, because of its large availability, well-known intercalation/exfoliation chemistry, high surface area and reactivity [11]. MMT comprises two tetrahedral silica sheets with an alumina octahedral sheet in the middle (2:1 layered structure), and the hydrated exchangeable cations occupy the spaces between lattices, as shown in Figure 7 [66].

#### 2.2.2. Metallic Nanoparticles

AuNPs, also called “gold colloids”, are the most stable among metallic NPs [67]. Generally, gold can be found in the Au^+^ (aurous) and Au^3+^ (auric) oxidation states. The synthesis of AuNPs involves reducing agents (e.g., citric acid, oxalic acid, hydrogen peroxide, borohydrides, polyols or sulfites), which act as electron donors that reduce Au^+^ or Au^3+^ to Au^0^. Afterward, stabilizing agents (e.g., trisodium citrate dihydrate; thiolates; and phosphorus ligands or surfactants, such as cetyltrimethylammonium bromide) are added in order to prevent aggregation and control NP growth in terms of rate, size and shape [68]. Recently, increased attention has been placed towards green synthesis methods that use plants, fungi and microorganisms, since their extracts are rich in natural reducing and stabilizing agents [68].

AgNPs are widely studied among metallic NPs due to their comprehensive application in fields such as medicine, pharmacology, microbiology, cell biology, food technology, water purification, house appliances and so forth [69]. They can be synthesized via sol-gel, hydrothermal, thermal decomposition, CVD, microwave-assisted combustion and biogenic synthesis methods. Their production involves reducing Ag^+^ to Ag^0^ using various biomolecules as electron donors, i.e., aldehydes, ketones, carboxylic acids, flavonoids, tannins, phenols and proteins [70].

CuNPs are naturally synthesized by plants via reducing Cu+ and Cu3+ ions. They can also be obtained by physical processes that require expensive instruments and chemical techniques, such as sonochemical reductions, thermal decomposition, electrochemical synthesis, hydrothermal processes, or microemulsions [71]. However, CuNP fabrication needs non-aqueous media and an inert atmosphere to prevent the formation of an oxide layer onto the surface. Other approaches include the protection of the NPs with capping agents or the conversion of CuNPs to CuO NPs.

#### 2.2.3. Metal Oxide Nanoparticles

ZnO NPs show great potential as antimicrobial agents owing to their large surface area, reduced size, high surface reactivity and ability to absorb UV radiation [72]. They can be synthesized through various methods, including thermal decomposition, combustion, vapor transport, the sol-gel method, the hydrothermal method, co-precipitation, ultrasonication and green synthesis using plant extracts or microorganisms.

TiO_2_ is an FDA-approved compound for food, drugs, cosmetics and food packaging usage [73]. It exists in three main polymorphs, namely, anatase, rutile and brookite [74]. The synthetic routes for TiO_2_ NPs include the sol-gel, hydrothermal and solvothermal methods, precipitation and electrochemical processes, using titanium chloride, titanium isopropoxide, or titanyl sulfate-based compounds as precursors. However, these techniques are detrimental in terms of reaction time and particle size control, hence novel green synthesis methods have emerged owing to their lack of toxicity and inexpensiveness.

Fe_3_O_4_ NPs have attracted a lot of interest for application within the biomedical field owing to their superparamagnetic and high magnetic susceptibility. Since their behaviour is strongly dependent upon their size, shape, structure, surface chemistry and colloidal stability, the choice of synthesis method is highly important. There are three main routes for Fe_3_O_4_ NPs synthesis, namely, physical, chemical and biological techniques, but the most commonly applied is chemical co-precipitation [75].

### 2.3. Organic Nanofillers

#### 2.3.1. Nanomicelles

**Figure 8:**
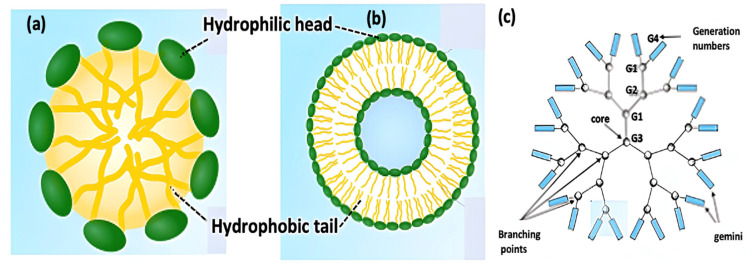
Representation of the structure of micelles (**a**), liposomes (**b**) and dendrimers (**c**).

Nanomicelles are formed via the self-assembly of amphiphilic molecules to form a globular structure with a diameter in the range of 5–100 nm (Figure 8a). The particles may be formed in aqueous or non-aqueous solutions in which the nonpolar region forms the interior and the polar region forms the exterior. Different surfactant molecules that may be non-ionic, ionic and cationic can be used to synthesize nanomicelles. They form only when the concentration of surfactant is higher than the critical micelle concentration (CMC), and the temperature of the system is greater than the critical micelle temperature or Krafft temperature. These two parameters are dependent on the amount of lipids and proteins in the micelles [76].

#### 2.3.2. Liposomes

Liposomes are small artificial vesicles, spherical in shape, and have at least one lipid bilayer (Figure 8b). Due to their hydrophobicity and/or hydrophilicity, biocompatibility and encapsulation capability, they are widely used for drug delivery [77]. Though liposomes can vary in size from several nanometres to a few micrometres, unilamellar liposomes are generally in the nanometre range, and can be prepared by sonicating a dispersion of amphipatic lipids, such as phospholipids, in water; novel methods such as extrusion, micromixing and the Mozafari method are employed to produce materials for human use.

#### 2.3.3. Nanodendrimers

Nanodendrimers are nanosized, radially symmetric molecules around the core, with a monodisperse structure that adopts a spherical three-dimensional morphology (Figure 8c). They are classified according to their generation, which refers to the number of repeated branching cycles that are performed during its synthesis. They have outstanding properties, such as polyvalency, self-assembling capability, good chemical stability, solubility and biocompatibility [78]. They are usually prepared via a divergent or convergent method. In both of them, the dendrimer grows outwards from a multifunctional core molecule. The core reacts with monomer molecules containing one reactive and two inactive groups, giving the first-generation dendrimer. Then, the new periphery of the molecule is activated for reactions with more monomers.

## 3. Machine Learning Applied to Polymeric Nanocomposites

ML is regarded as a subset of artificial intelligence that is mainly concerned with the development of algorithms, which allow a computer to learn from the data and past experiences on its own. In 1950, Alan Turing (considered the father of artificial intelligence) published a paper entitled “Computer Machinery and Intelligence”, on the topic of artificial intelligence. In this paper [79], he posed the question “Can machines think?” In 1952, Arthur Samuel, who was the pioneer of machine learning, created a program that helped an IBM computer to play a checkers game. In 1959, the term “Machine Learning” was first coined by this author, who defined it as “a field of study that gives computers the ability to learn without being explicitly programmed”. It drew the attention of many scholars who began investigating this area. In 1959, the first neural network was applied to a real-world problem to remove echoes over phone lines using an adaptive filter. Progressively, ML turn out to be a stirring tool for the scientific community, since various statistical and probabilistic methods were demonstrated to speed up both fundamental and applied research [80]. ML algorithms have been widely applied in the fields of biology and chemistry [81,82], which has stimulated material researchers to explore the option of using them for the design of novel materials with improved properties and wider applications [83]. The combination of experiments and computer simulations produces a huge amount of data that enable us to integrate ML algorithms with material science for property prediction and novel material design. In the following sections, the applicability of ML algorithms to polymeric nanocomposites is described.

### 3.1. Classification of Machine Learning

At a broad level, machine learning can be classified into three types: (1) supervised learning; (2) unsupervised learning; and (3) reinforcement learning. In supervised learning, sample labelled data are provided to the machine learning system in order to train it, and on that basis, it predicts the output [84]. In other words, an algorithm is used to learn the mapping function from the input to the output: y = f(x) [85]. The goal is to approximate the mapping function so well that when new input data (x) are provided, the output variables (y) for that data can be predicted. Once the training and processing are completed, the model is tested by providing a sample data to check whether it is predicting the exact output or not. It is named as “supervised learning” since the process of an algorithm learning from the training dataset is comparable to a student learning under the supervision of the instructor. The learning stops when the algorithm attains a satisfactory level of performance. It can be further divided in two categories of algorithms: classification and regression. In a classification problem, the output variable is a category, such as “red” and “blue” or “disease” and “no disease”. In a regression problem, the output variable is a real value, such as “dollars” or “weight”. Typical examples of supervised learning algorithms are linear regression, random forest, spam filtering and support vector machines [85].

In unsupervised learning, a machine learns without any supervision. The training is provided to the machine with the set of data that has not been labelled, classified or categorized, and the algorithm needs to act on that data without any supervision [86]. The aim is to restructure the input data into new features or a group of objects with similar patterns. There is no predetermined result. The machine tries to find useful insights from the huge amount of data. It can be divided into two types of algorithms: clustering and association [87]. A clustering problem is when you want to discover the inherent groupings in the data, such as grouping customers by purchasing behaviour. An association rule learning problem is when you want to discover rules that describe large portions of your data, such as people that buy X also tend to buy Y. Some popular examples of unsupervised learning algorithms are k-means for clustering problems and the apriori algorithm for association rule learning problems.

Reinforcement learning is a feedback-based ML method, in which a learning agent gets a recompense for each correct action and a penalty for each mistaken action. The agent learns automatically with this feedback and enhances its performance. The aim is to obtain the most reward points. In reinforcement learning, the agent interacts with the environment and explores it. The robotic dog, which automatically learns the movement of his arms, is an example of reinforcement learning.

**Figure 9:**
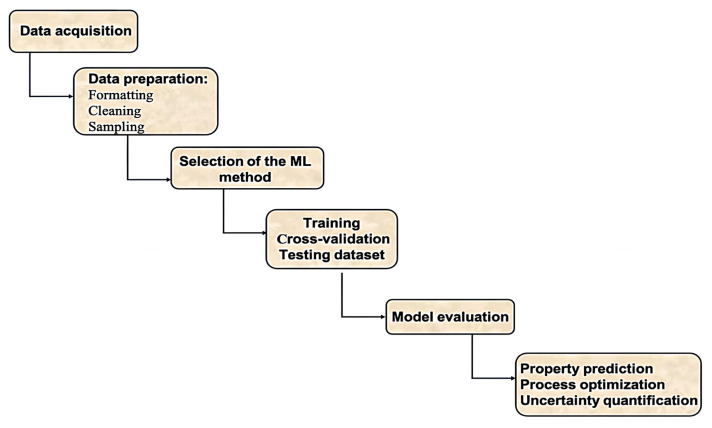
Scheme of the six-stage approach for the implementation of ML algorithms.

Among the three abovementioned types, supervised learning is the most commonly used in the field of polymeric nanocomposites. Algorithms that fall under this category typically follow a six-stage approach as described in Figure 9.

**Figure 10:**
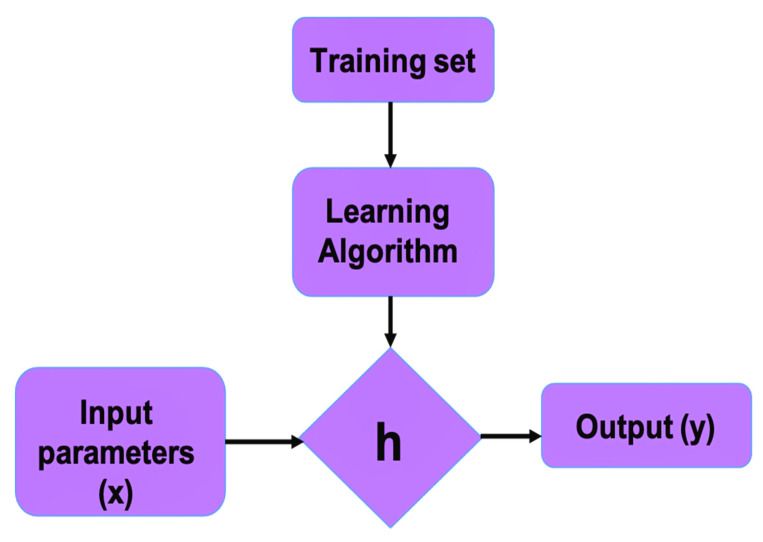
Basic scheme for initial implementation of ML algorithms (h: x→y) where h represents a hypothesis function that maps the input parameters (x) to the output (y) and selects a suitable learning algorithm to be used for further prediction.

- Data acquisition: data should be collected in a systematic way from published articles, technical reports or from own experimental data. It should be noted that some data sources or published articles do not report all considered variables.
- Data preparation: After collecting the suitable data, preprocessing is carried out in terms of formatting, cleaning and sampling. Formatting provides a structure to the data which enhances its quality. Relevant materials and process variables affecting the behaviour to be modelled need to be carefully examined. Nanofiller-related parameters need to be considered, such as type, concentration, shape and size, matrix parameters including nature and concentration, the manufacturing process to fabricate the nanocomposites as well as some nanocomposite properties, such as density, thickness, porosity, etc. Some of the attributes are deleted in the cleaning step in order to keep the consistency of all recovered values and ensure data quality. Incorrect data can hinder the accuracy of ML predictive models. Erroneous data may arise during both recovering data points from the literature or entering the datapoint into the database. All entered values need to be double checked to verify that no erroneous value was included. Then, sampling is used to select a subset of the data out of a big chunk which can further be used for the training purpose [88]. Converting the raw information into certain relevant attributes which are further used as input features for the selected algorithm is a necessary step for getting accurate predictions and is commonly known as feature engineering [89]. It helps in increasing the learning accuracy along with improved comprehensibility.
- Selection of the ML method: After data preparation, the next step is to set a hypothesis function (h(x)) which maps the input parameters (x) to the output (y) and selects a suitable ML algorithm to be used (Figure 10). Based on the type of data and whether the problem is a classification or regression, an appropriate algorithm is chosen [90]. The algorithms most widely used for classification problems are K-nearest neighbour (KNN), decision trees, neural networks, naive bayes and support vector machine [91]. For problem regressions, algorithms such as linear regression, support vector regression, neural networks, Gaussian process and ensemble methods are typically applied [92].
- Training: The selected algorithm is trained with the processed data, which are split into three subsections: training, cross-validation and testing dataset. The model learns to process the information using a training dataset. A cross-validation dataset is used for parameter tuning and to prevent overfitting issues.
- Model evaluation: This is a critical part of the model development process. A model can be inaccurate despite having a very small data training error. With this aim, a test dataset is applied to assess the model’s performance, and this sets the basis for making the final predictions. Accordingly, the final model is chosen and the hypothesis function is also evaluated. Figure 10 shows the basic scheme for the initial implementation of ML.

### 3.2. Property Prediction, Process Optimization and Uncertainty Quantification

**Figure 11:**
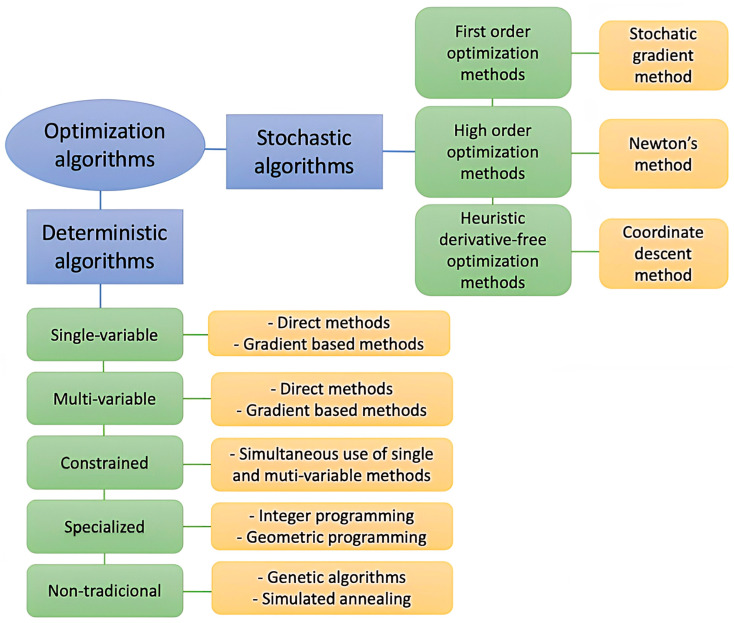
Classification of optimization algorithms based on the design variables, objective function and type of constraints.

ML has been applied to polymeric nanocomposites for the prediction of the material properties, process optimization, microstructural analysis and the quantification of uncertainties arising in the material and its properties due to the complex manufacturing processes. Optimization is one of the most important applications of ML. It involves the process of training different models multiple times, which is computationally very expensive and has the tendency of becoming intractable for complex simulations. An optimization algorithm carries out iterative execution by comparing different models for potential solutions until a satisfactory result is found. Three basic keys define an optimization problem: (1) variables, the parameters the algorithm can tune; (2) constraints, the boundaries or limits for these parameters; and (3) the objective function, the goal towards which the algorithm progresses. Figure 11 displays the classification of optimization algorithms based on the design variables, objective function and constraints.

Two types of optimization algorithms can be applied: deterministic, which make use of specific instructions to find the solution and the uncertainties in terms of variable space are ignored [93,94]; and stochastic, which are probabilistic methods wherein the uncertainties are modelled with suitable probability distributions [95]. A novel approach, named robust optimization, is also used to explicitly model and minimize the uncertainty involved in the problem by using a set-based deterministic description of the uncertainties [96].

**Figure 12:**
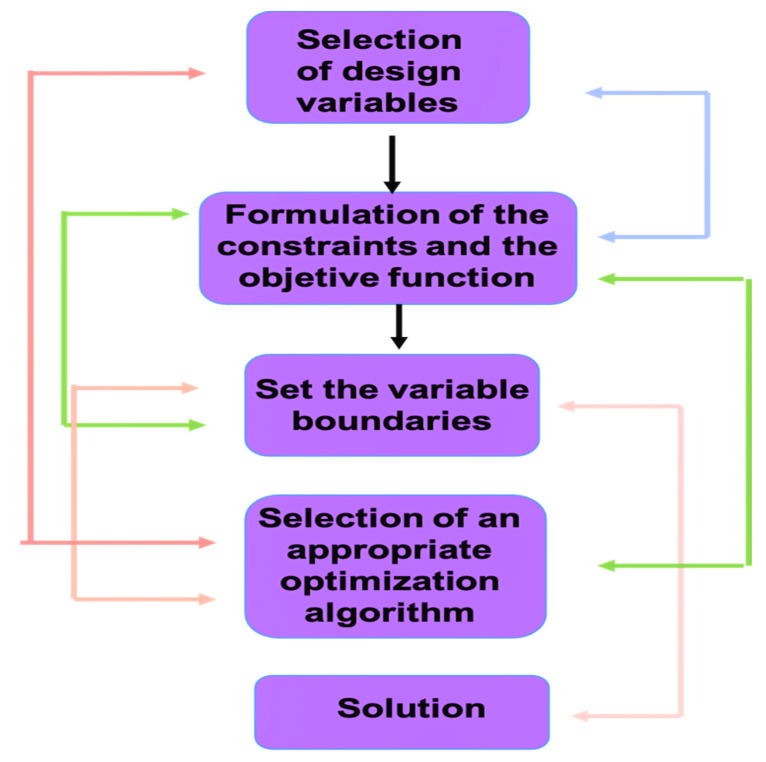
Flowchart for the optimal design. An optimization procedure can be used to obtain optimal solutions concerning various types of engineering problems for choosing the right combination of input parameters.

Stochastic algorithms use random objective functions and constraints for problem optimization. Optimal design is attained by comparing different potential hypothesis functions and then estimating each of their corresponding cost function (squared error function) by identifying the design variables and constraints. The whole optimal problem is then expressed in a mathematical form and is solved using an optimization algorithm. A scheme of the procedure followed for the design of an optimal problem is shown in Figure 12.

**Figure 13:**
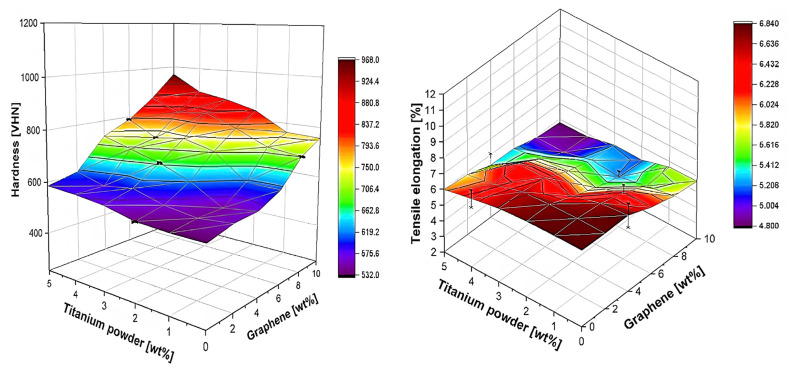
Prediction results of hardness and tenisle elongation of PEEK/C/Ti hybrid nanocomposites. Reprinted from Ref. [101], copyright 2022, with permission from Elsevier.

Optimization methods can be significantly improved in terms of efficacy and efficiency by involving ML algorithms [97,98]. For instance, Salah et al. [99] optimized the process parameters and predicted the absorption index of polycarbonate (PC)/CNT nanocomposites using a ML of multilayer perceptron network approach. Khanam et al. [100] optimized the thermal conductivity, crystallization temperature, degradation temperature and tensile strength of linear low-density polyethylene (LLDPE)/graphene nanoplatelets (1–10 wt%) nanocomposites processed in a twin-screw extruder with three different screw speeds and feeder speeds of 50, 100 and 150 rpm. The prediction of properties was performed via an artificial neural network (ANN). The first three properties increased with rise in both screw speed and graphene content. The tensile strength reached a maximum at 4 wt% and a speed of 150 rpm, and these were the optimum conditions for the stress transfer from the amorphous chains of LLDPE to the graphene nanoplatelets. A similar approach was used by Zakaulla et al. [101] to predict the mechanical properties of high performance polyetheretherketone (PEEK) hybrid nanocomposites comprising graphene (2–10 wt%) and titanium powder (1–5 wt%) prepared via injection moulding [101]. The proposed ANN model delivered satisfactory results to predict the hardness, tensile strength, modulus of elasticity and tensile elongation in comparison to experimental measurements (Figure 13), and the best performance was attained upon the incorporation of 10 wt% graphene. The correlation factor connected with the training and test dataset was greater than 0.9.

Yusoff et al. [102] predicted the rheological properties of nanosilica/polymer modified bitumen using multilayer perceptron neural network models, and attained very good agreement with the experimental data with R value of 0.978. Recently, Kosicka et al. [103] used different optimization algorithms to predict the mechanical properties of epoxy-based nanocomposites reinforced with alumina in the concentration range 5–25 wt%. By using the Python programming language and available libraries, a neural network generated the predicted values of selected properties of the nanocomposites, including Young’s modulus, maximum stress, maximum strain and hardness. The comparison of forecast values with the values obtained at the stage of laboratory tests confirmed the effectiveness of the network (63% of forecasts were classified as very accurate, 15% of forecasts were defined as accurate).

Computational analyses of polymer nanocomposites often meet uncertainties because of the variations in the material properties, measurement uncertainty, restrictions in the test set-up, operating environment and inaccurate geometrical features [104]. Uncertainty in parametric inputs, initial conditions and the boundary conditions, computational and numerical uncertainties arising from the unavoidable assumptions and approximations together with the intrinsic inaccuracy of the model lead to major deviation from the deterministic values or the expected material behaviour, altering the overall nanocomposite performance. To ensure that the simulation results are reliable and to understand the risks for making final product decisions, it is crucial to quantify these uncertainties [105]. In this regard, Doh et al. [106] used the Bayesian inference approach to quantify the uncertainty of percolating the electrical conductivity of polymer/CNT nanocomposites. The correlation between the CNT conductivity and the phase transition parameter along with the critical exponent significantly affects the electrical conductivity of the resulting composite in the uncertainty quantification.

### 3.3. ML Algorithms Used in Polymer Nanocomposites

With the aim to optimize design, researchers are continuously investigating the exploitation of the growing capabilities of ML algorithms. Such research activities have resulted in many successful attempts which are summarized in the following subsections.

#### 3.3.1. Neural Networks

**Figure 14:**
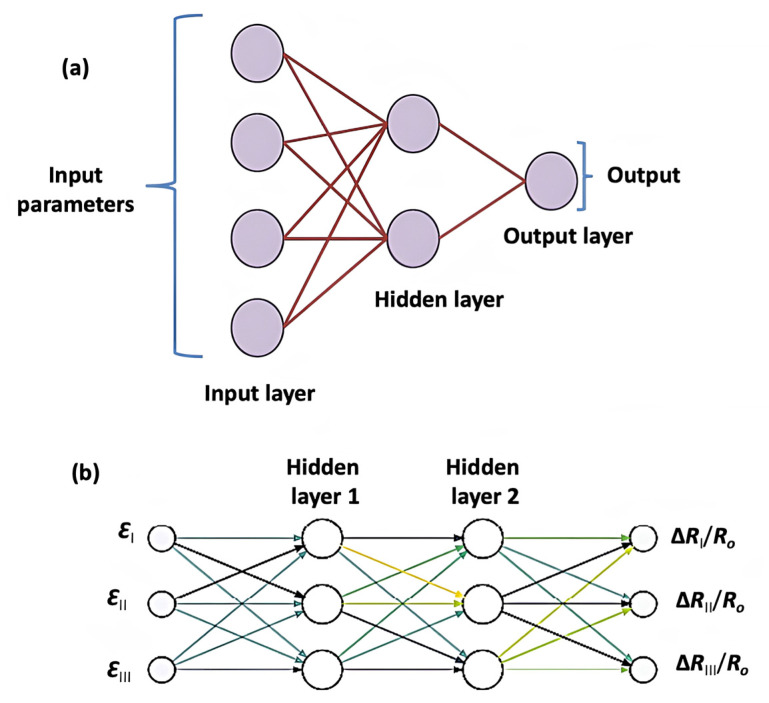
(**a**) Scheme of the artificial neural network (ANN) basic architecture with input, hidden and output layers to receive the preprocessed data, compute the correlations and give the predicted output, respectively. (**b**) ANN for predicting the electro-mechanical response of polymer/CNT nanocomposites.

Neural networks are the favourite algorithm of material science researchers to investigate data-intensive aspects. They are mathematical tools inspired by the biological nervous system and are used to solve a wide range of problems by recognizing underlying relationships in the available data [107]. In the human brain, there are millions of neurons connected via a network which aids in processing the flow of information to generate meaningful outputs. Similarly in neural networks, there are number of neurons that act as processors operating in parallel and arranged in different layers. The first layer (input layer) collects all the information to be considered (preprocessed data). Then the intermediate (hidden layer), comprising many discreet nodes, is responsible for all the computations [108]. The last layer (output layer) provides the final predictions. A scheme of the basic architecture of an artificial neural network (ANN) is provided in Figure 14. An ANN was defined by Aleksander and Morton [109] as a massively parallel-distributed processor made up of simple processing units, which has a natural propensity for storing experimental knowledge and making it available for use. It is similar to the brain in two aspects: (1) Knowledge is acquired by the network from its environment via a learning process; (2) Inter-neuron connection strength, known as synaptic weights, is used to store the acquired knowledge.

The most suitable applications of ANNs are those that have a large available dataset, in which it is difficult to find an accurate solution due to the existence of several mathematical approaches and when the dataset is incomplete, noisy or complex. Some properties of polymer nanocomposites, such as fatigue, wear, creep, etc., are suitable for ANN analysis [110]. It is ideal in polymeric nanocomposites when only the material composition and testing conditions are the input data. It can aid to simulate the relationship between the manufacturing parameters and the material performance, which can be used as the basis for a computer processing optimization. The required number of trained data can be reduced by optimizing the ANN architecture and by choosing suitable input parameters. Multilayer perceptrons (MLPs) and radial basis functions (RBFs) are predictor functions frequently used in ANNs [111] which help to minimize the error in the predicted outputs.

Feedforward (FF) architecture with backward propagation (BP) is typically applied for output computation and error minimization. In the FF style, no loops are formed in the whole network. Information in any of the units of the successive layers does not receive any feedback, while in the back propagation, synaptic weights are adjusted by back propagating the error. Weights are updated after each record is run through the network. One iteration is completed when all the records finish running through the network and it is known as epoch. The process is repeated after completing one epoch. There are mathematical equations (activation functions) for linking the weighted sums of each layer with the succeeding layer and delivering the output.

The architecture of ANN model can be mathematically expressed as:

*Y* = *F* (*x*) *Y* = *A* (*W* × *x* + *B*_i_) + *B*_0_

where *Y* is the output vector, *x* is the input vector, *A* is the activation function (sigmoid, Tanh, softmax, softplus etc.), *W* is the matrix that contains the synaptic weights, *B_i_* is the column vector of biases from the input layer to the hidden layer and *B*_0_ is the column vector of biases from the hidden layer to the output layer.

**Figure 15:**
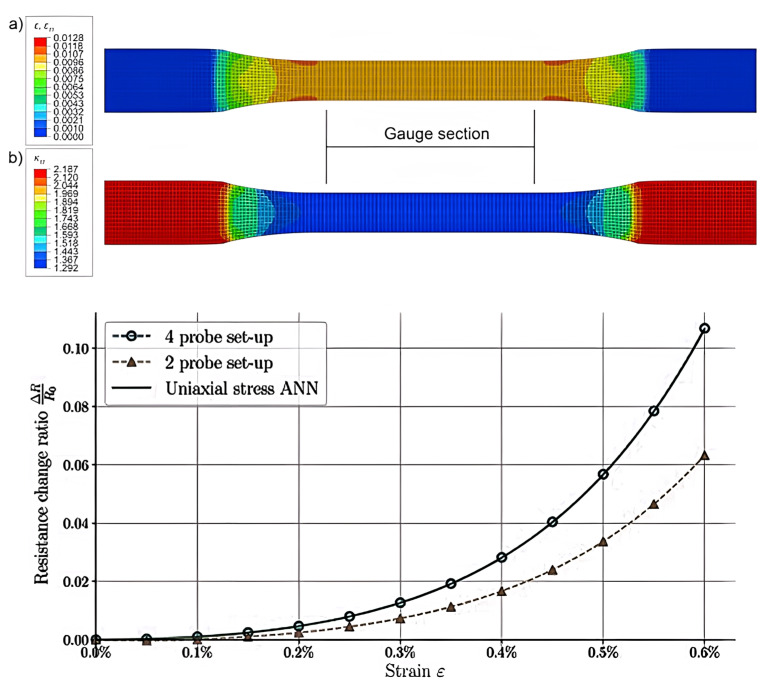
Application of ANN to epoxy/CNT nanocomposites for the manufacture of damage detecting sensors. Top: Contours of the (**a**) axial strain and (**b**) axial conductivity fields when a strain of 1% acts in the gauge portion. Down: measured changes in resistance for the 2-probe and 4-probe setups, compared with the expected resistance versus strain history predicted in the case of uniaxial stress (by the ANN). Reprinted from Ref. [112], copyright 2019, with permission from Elsevier.

Matos et al. [112] applied an ANN to examine the potential of epoxy/CNT nanocomposites for the manufacture of damage-detecting sensors. The finite element method (FEM) was used to produce extensive data for ANN, which was then used, at a macroscopic scale, to predict the conductivity of the nanocomposites as a function of multiaxial strain up to 1% (Figure 15). Flat dog-bone specimens, dimensioned according to ASTM D638-02, were used to carry out measurements of the strain-sensing response [112]. Such measurements occur by placing two electrodes at opposite ends of the specimen end tabs; a potential difference can then be measured by the same electrodes (2-probe setup) or by an additional pair of electrodes at different locations along the specimen (4-probe setup). A volume fraction of 1.0% was selected since it is just above the percolation threshold, and in this region the nanocomposites are expected to display the most prominent strain-sensing response. The elastic modulus of the CNTs and epoxy matrix, Poisson’s ratio, CNT diameter and length were input parameters. The approach did not require calibration parameters to be determined from complex experiments and took into account the quantum tunnelling electron transport at the junction between CNTs. The conductivity along the specimens was non-uniform and its value in the gauge portion was approximately 50% of the value at the undeformed end tabs, suggesting that a 4-probe setup should yield much more accurate measurements than a 2-probe setup (Figure 14). A chief result of this work was the decrease in computational time: simulations carried out with the FEM needed 3.5 h to run, while the ANN obtained the same output in less than 0.2 s.

Different variants of neural networks have been successfully used to predict the behaviour of polymeric nanocomposites under different conditions, and some representative studies are summarized in Table 1. Adaptive neuro fuzzy interference systems (ANFISs) are another algorithm that has the benefits of neural networks as well as fuzzy logics and integrates the principles of both in a single architecture. Researchers have implemented ANNs and ANFISs to predict the impact strength, yield strength and other mechanical properties of polymeric nanocomposites [113], and concluded that both can be successfully implemented to any type of polymer composite to predict mechanical behaviour. Another class is a convolutional neural network (CNN) which falls under the category of deep learning. Researchers have applied it for analysing images and quantitatively predicting the mechanical behaviour of composites by making use of different grid sizes of the composite microstructure obtained from scanning electron microscope (SEM) analysis. Using the chemical structure of different polymers, it possible to predict the glass transition temperature (T_g_) along with other polymeric properties.

#### 3.3.2. Genetic Algorithm

Another approach that can be used to predict the properties of polymer nanocomposites is the genetic algorithm (GA). Since its origin in 1975 by researcher John Holland, the genetic algorithm [137] has been widely studied and developed. It is a bio-inspired algorithm based on the theory of evolution of species [138], in which individuals better adapted to their environment have a better chance of surviving and having offspring, thus transmitting their genetic characteristics to future generations. In this comparison, each individual represents a solution to the optimisation problem to be solved and the genetic load of the individual is precisely the value of the parameters that define the encoding of the solution. In the design of polymeric nanocomposites, each individual in the population encodes a specific nanocomposite design. The algorithm manages simultaneously with a population of individuals competing to reach the next generation (parallel algorithm) [139]. It is defined as an evolutionary algorithm because its execution is based on the evolution of successive stages for the population of solutions. The fact that it speaks of the probability of an individual’s survival depending on the degree of adaptation makes the algorithm non-greedy: factors other than the genetic load itself, such as fortuitous circumstances, may favour an individual to thrive until a later generation.

The adaptation of an individual is measured by an evaluation function or fitness function. The fitness function corresponds to a measure of one or more desirable properties in the nanocomposite. A better quality in such properties means a higher probability of survival among the population. Depending on the problem, optimisation consists of either maximising or minimising this function. In the design of nanocomposites, the goal may be to maximize the material strength, the thermal conductivity, the electrical conductivity [140] or the hydraulic performance [141]. Other cases are aimed at minimizing the cost function, in [142] the potential energy of Au-Ag bimetallic nanoparticles is minimized, in [143] the friction factor is minimized while the heat transfer is maximized. Otherwise, the goal is the classification of the samples according to their structure [144], so the fitness function is defined as the classification accuracy, which needs to be maximized. The evaluation function makes it possible to discriminate between individuals by determining which of them is the best adapted, i.e., the one with a fitness value closest to the maximum/minimum of the function.

The implementation of a genetic algorithm is characterised by three basic operators [145]: the selection operator for selecting individuals to compose the population of a generation and/or determine the individuals that will have offspring, the crossover or recombination operator for obtaining individuals descended from progenitor individuals, and the mutation operator, which, with reduced probability, causes substantial changes in the composition of some individuals. The consequences of mutation in an individual can be disastrous for its survival or, on the contrary, very advantageous for it.

**Figure 16:**
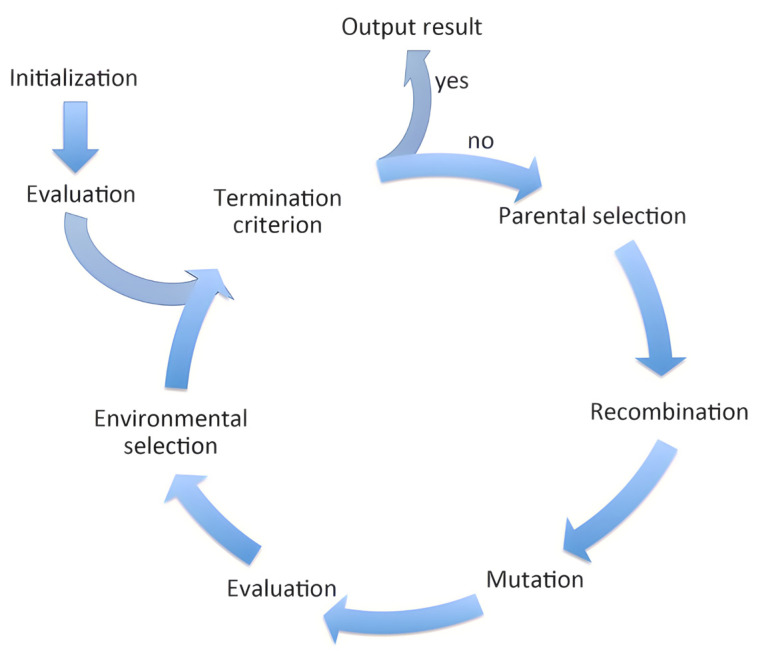
Flowchart of a basic genetic algorithm.

Figure 16 shows the basic flowchart of a genetic algorithm. The algorithm starts with a set of random individuals that make up the initial population. The initial population represents a set of random solutions to the problem. Since these are random, they are expected to have fitness values far from the optimum. If the termination conditions of the algorithm are not met, the algorithm progresses to the next generation. In each generation, the population is processed with the parent selection, crossover and mutation operators. The new individuals are then evaluated and the selection of survivors for the next generation is performed. If the stopping conditions are not met again, a new generation is started.

This basic diagram accepts additional procedures, such as individual repair and/or local search. Individual repair is necessary when the encoding of the solutions allows an individual not to correspond to a feasible solution. It is then necessary to repair the individual so that it represents a single feasible solution. The local search process consists of making small changes to the individual so that it results in another individual that is very similar to the first one, but of higher quality. The local search operation means making a small shift in the solution space, as opposed to mutation which is a more distant jump in the solution space. The local search function significantly increases the computational cost, so it is usually introduced only in some cycles of generations. Including such techniques in the genetic algorithm makes the algorithm a hybrid method.

The stopping or termination criteria of the algorithm are generally defined as one or more of the following:

- The evolution of a maximum number of generations.
- Reaching a certain number of generations in which no appreciable improvement in the population is detected. After successive generations the average fitness value in the population remains constant.
- Finding a solution sufficiently close to a previously bounded optimum.

In the following, we discuss in detail the three basic operators mentioned along with the encoding of the solution and the local search process. A more detailed explanation of the theory of genetic algorithms can be found in [146,147,148,149].

##### Solution Coding

**Figure 17:**
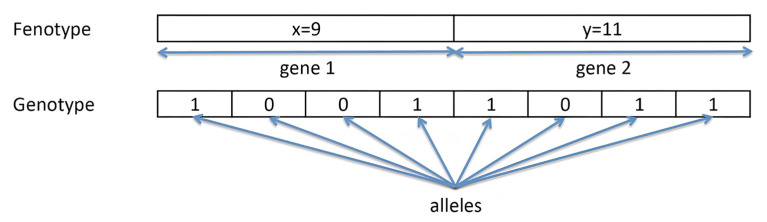
Example of binary coding. The coding of the solution is known as the genotype. The individual or chromosome is composed of a given number of genes. The phenotype corresponds to the solution represented by the chromosome.

The encoding process allows the representation of the solutions of the real problem in individuals manageable by the algorithm. Each individual is represented in the form of a numerical vector called a chromosome. Each element of the vector is called an allele. A set of alleles encoding the same characteristic or solution parameter is called a gene. Figure 17 shows an example of binary coding, in which the chromosome (10011011) represents the solution {x = 9, y = 11}. The chromosome is composed of two genes (gene (1001) refers to the variable x and the second half of the chromosome or gene (1011) corresponds to the variable y). Each bit that makes up a gene is an allele.

Genotype or genotypic space is defined as the space of individuals, while the phenotype or phenotypic space is the space of solutions to the real problem. In the example of Figure 17, the vector (10011011) corresponds to the genotype, while the solution {x = 9, y = 11} refers to the phenotype.

There are encodings based on order, in which the order that the variables appear in has a meaning in the encoding of the solution. These include coding by permutations, as in the case of the Travelling Salesman problem, and other codings used, for example, for solving Sudoku puzzles.

In other encodings, the order of the variables is not important for the solution itself, which is identified by the value of each gene. There are four types of non-order-based encodings:

- Binary coding. This can be either common binary coding or Gray’s binary coding.
- Coding with integer values, the content of the genes belongs to the set of integers ℤ.
- Coding with real values, analogous to the previous one but the variables can only take values in ℝ.
- Finite value coding, in which the variables can take only values pertaining to a limited set of values, such as a set of predefined colours or a closed set of positions on a board.

The coding method chosen for a genetic algorithm greatly influences the ability of the genetic algorithm to find high quality solutions [150]. It especially affects the parameter called locality. The level of locality is closely related to how a solution (phenotype) is altered by applying small changes to its chromosome (genotype). A higher level of locality implies a lower degree of modification of the solution following changes made to the individual.

##### Selection of Individuals

The process of the selection of individuals takes place both in the composition of the population for the next generation and in the step prior to the recombination of individuals. The selection of individuals, the form and the percentages of the total population are defined in the architecture of the algorithm. In some cases, not all individuals participate in recombination. In others, an individual is allowed to be selected several times as a parent in different pairs.

The selection of individuals is based on the fitness value of them, so those with better fitness values, i.e., better adaptation, are given a higher probability of selection. However, randomness does not prevent some poorer quality individuals from passing the selection process. The efficiency of a selection operator is measured according to its degree of selective pressure and its degree of diversity, both of which are opposing factors:

- Selective pressure: allows the best individuals to be selected for the recombination process. It is necessary so that the search process is not random and there is a certain degree of convergence, focusing the search on promising regions.
- Diversity refers to the differences between individuals. The lack of genetic diversity causes all individuals in the population to be similar, so their offspring will be similar as well. The algorithm will progress very slowly or not at all.

Selection techniques can be classified into three main groups [151]: tournament selection, uniform state selection and proportional selection:

- Selection by tournament: this technique was proposed by [152] and subsequently studied in [153]. Selection is performed by direct comparisons of the fitness values of individuals. A number p of individuals is randomly selected. Typically, p = 2 is used, i.e., a tournament by pairs of individuals. From each pair, the individual with the best fitness wins. This process is repeated until the selection of individuals is complete. A variant of this method is to assign a probability of success to the fittest individual in each set. In this way, the fitness value does not always win.
- Uniform state selection: was proposed by [154] for non-generational genetic algorithms, in which in each generation, only a few individuals are replaced by fitter ones. It consists of selecting the fittest individuals and subjecting them to the crossover and mutation operators. The resulting fittest offspring will replace the worst individuals in the population in the next generation.
- Proportional selection: individuals are chosen according to the contribution of their fitness value with respect to the contributions of the rest of the population. This technique was originally presented by [137] and further developed by [155]. Variants have appeared, the most widely used of which is the roulette technique.

The roulette selection method consists of assigning each individual a probability of selection proportional to the position of its fitness in the interval of fitness values of the existing population. In this way, individuals with better fitness have a greater chance of being selected than individuals with worse values of the evaluation function. This technique has become very popular since it was published in [148]. In the following, we show a numerical example of selection using the roulette technique. Let us suppose that the algorithm tries to maximise the cost function in a population composed of six individuals (*N_ind_* = 6) with identifiers from 1 to 6. Table 2 records them in a decreasing order according to their fitness F*_i_*. The first column of the table indicates the identifier of each individual *i*, while the second column gives its fitness value F*_i_*. The sum of all fitness values in the population is 20 as Table 2 shows in the last row. We set the fitness range [1,2,3,4,5,6,7,8,9,10,11,12,13,14,15,16,17,18,19,20], which will be proportional to the selection probability range [0,1]. The third column of the table indicates the selection probability *p_i_* of individual *i* by the following equation. The individual with the best fitness has a probability 1 of being selected in the operation (crossover or for the next generation), which is an elitist behaviour. As the fitness value gets worse, individuals have lower probability of selection. The individual with the worst fitness has a very small, but non-zero probability, allowing the algorithm a certain randomness or chance in the continuity of poorer adapted individuals.

$$p_{i}=\{\begin{array}{c} \frac{F_{i}}{\sum_{i=1}^{N_{ind}}F_{i}};i=1\\ p_{i-1}+\frac{F_{i}}{\sum_{i=1}^{N_{ind}}F_{i}};1<i\leq N_{ind} \end{array}$$

When the goal is to minimize the cost function, Table 2 is built by sorting the individuals in decreasing order of fitness.

##### Crossing Operator

**Figure 18:**
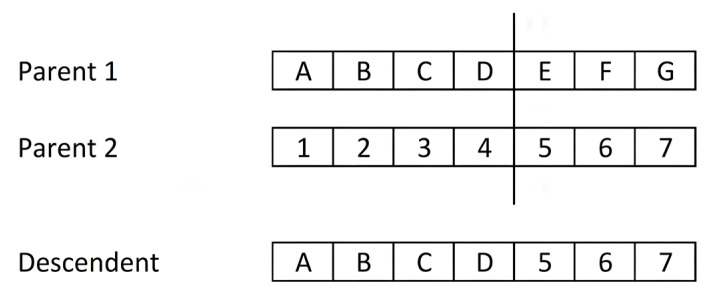
Example of the crossover operation with single point cutting in a genetic algorithm. Each chromosome is fragmented into two parts and the offspring receives one fragment from each parent.

The aim of the crossover or recombination operation is to explore the solution space in an intelligent way. A new chromosome or individual is generated from two of them. In the composition of the offspring, the aim is to extract, preserve and combine part of the genetic material of its parents, while at the same time adding new sections to the chromosome that bring diversity to the population. There are numerous types of crossbreeding, some of which will be briefly described here:

- Single point crossover: This method was proposed by [137] and is the simplest and most popular. Subsequently, several variants have emerged. It consists of sectioning both parent chromosomes at a random point, with each chromosome being subdivided into two parts. The offspring is composed of one part of each chromosome. Figure 18 shows an example of a single-point crossover. We have used letters (A–G) for the content of the first parent and numbers (1–7) for the second, with the intention of highlighting the origin of the fragments in the offspring. Both parents have a length of seven alleles, and the cut-off point is located between the fourth and fifth allele. The offspring is assembled from the initial fragment of parent 1 and the final fragment of the chromosome 2.

Depending on the coding used, sometimes not all points on a chromosome are suitable as cut points. This is the case of cutting at an intermediate point of a gene, possibly generating offspring with an invalid gene value. The repair function is responsible for solving conflicts in the viability of offspring.

**Figure 19:**
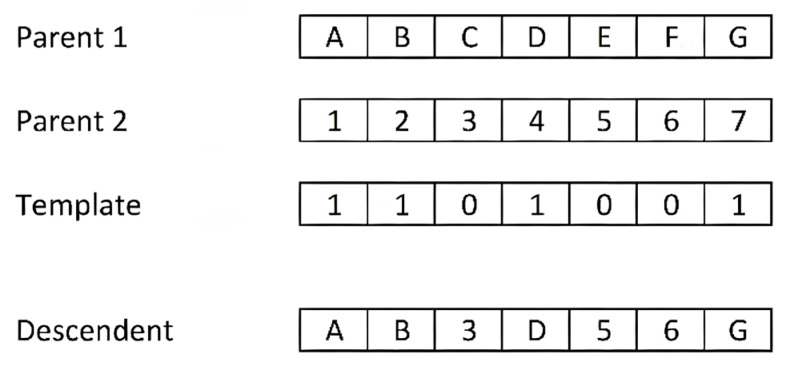
Example of the uniform crossover operation in a genetic algorithm. The descendant chromosome is assembled by taking genes from either parent as indicated by the binary mask.

**Figure 20:**
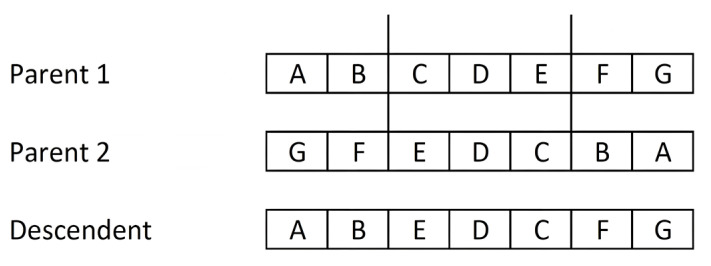
Example of a partial map crossover in a genetic algorithm. The offspring chromosome takes up complete fragments of one of the two parents, filling in its gaps with the genes still not present and in the order of appearance in the second parent.

- Multi-point crossover: this was proposed by [156] as a generalisation of single-point crossover. It consists of fragmenting both of the parent chromosomes by N random points, thus obtaining N + 1 fragments of each. The offspring is built by alternating fragments from each parent. It has been experimentally proven that the N = 2 value gives better results than single-point cutting. This method shows a greater tendency to fragment the chromosomes in the central sections than in the areas near the ends [157].
- Uniform crossover: this can be considered as the extreme case of N-point crossover in the sense that each gene is considered a fragment of the chromosome. The offspring is formed by permuting the genes of both parents with a certain probability. In most cases a probability of 0.5 is taken, although some researchers recommend a somewhat lower probability. The assignment of the content for each gene of the offspring is performed according to a binary mask of the same length as the parent chromosomes, in which the value “1” at position i means assigning gene i of the offspring the value of the same gene of one parent, while the value “0” indicates assigning the content of gene i of the other parent. Figure 19 shows an example of a uniform crossover with a binary mask generated by applying a probability of 0.5. Therefore, the offspring receive information from both parents at 50%.
- Shuffle crossover: this is a technique that can be incorporated into the three previous types of crossovers in order to reduce the tendency to fragment chromosomes at the central sections. It consists of applying the same random permutation to both parents before the crossing operation. After the crossover operation, the reverse permutation must be applied to the offspring. In this way, the positions of the cut-off points are more evenly distributed throughout the individual.
- Partial map crossover: this operator is applied in order-based encodings (permutations) in which the value for a gene cannot be repeated in the same chromosome. The classic example for this type of coding is the Packet problem, in which each packet is represented only once in the chromosome content. This type of crossover copies part of the genetic information of one of the two parents into the offspring, with its exact sequence and in the same position. The crossover process is as follows: N cut points are applied to one of the parents and alternating fragments of the parent are transferred in their entirety to the individual offspring in a similar way to the multipoint crossover. For N = 2, the first and third fragments of the selected parent would be copied to the offspring. The remaining fragments for the offspring are filled with the values of the genes not present in the offspring and in the order of occurrence of the second parent. Figure 20 shows an example of this method with N = 2 and the cut-off points between the second and third genes, and between the fifth and sixth genes. We have chosen the parents (A B C D E F G) and their inverse (G F E D C B A) to have a simple example. The offspring chromosome takes the end fragments from the first parent, resulting in (A B - - - F G). We filled in the three free gaps with the genes from the second parent in order of appearance, if not already present. In the example, the first two genes of the second parent (G F) are not considered because they are already included in the offspring; only (E D C) is filled in.

##### Mutation Operator

The mutation operator is generally applied on a small percentage of the population. However, mutation effects have a great influence on the evolution of the algorithm. In fact, some evolutionary algorithms, although not genetic algorithms, use mutation as a fundamental strategy in the search for solutions. The idea behind this operation is to recreate the genetic mutations produced in species in nature, due to errors in DNA transfer. In genetic algorithms, mutation achieves several objectives:

- The exploration of new areas in the space of solutions close to “quality solutions” already studied.
- Ensures diversity in the population to avoid premature convergence of the algorithm.
- In case of premature convergence to a local optimum, the algorithm can be free from that local maximum/minimum.

The mutation operation is applied after the crossover operator and only to a defined percentage of the population. This percentage usually varies between 1% and 5% for binary coding, and no more than 15% for real coding. Too large a percentage of the mutated population can turn the search for solutions into a virtually random search. Meanwhile, too small a percentage would not help the algorithm to get away from possible premature convergence.

To prevent the best individuals in the population from being mutated with poorer quality results, one or more copies of these individuals are usually conserved to avoid losing them. There are different types of mutation depending on the type of coding used. In binary coding problems, binary mutation is basically applied. This method is the simplest and most popular.

In binary mutation, a random binary mask is used. The genes of the individual that have the same position as the “1’s” in the template are modified, i.e., the “1’s” of the individual become “0’s” and vice versa. The genes in the positions in which the mask contains “0” are not altered.

A variant of the binary mutation is the uniform binary mutation. The special feature is how the template binary mask is constructed. A random vector of real numbers in the interval [0,1] of the same length as the chromosome to be mutated is generated and a probability threshold is defined. Mask positions matching a value in the vector greater than the threshold write a “1” in the mask, and “0” the vector value is less than or equal to the threshold.

For real, integer or finite coding, we have non-uniform mutation and modified non-uniform mutation. The non-uniform qualifier refers to the fact that the probability of mutation is variable, depending on the number of generations elapsed. The probability of mutation decreases as generations progress. This aspect is similar to the simulated annealing process, in which the system freezes as the algorithm progresses. The following equation allows us to calculate the mutation probability *p_i_* in generation *i*, always variable within the range [*p_Min_* − *p_Max_*], where *p_Min_* is its minimum value and *p_Max_* indicates the maximum mutation probability. *N_Max_* corresponds to the maximum number of generations the algorithm can run.

$$p_{i}=p_{Max}-\frac{i\left(p_{Max}-p_{Min}\right)}{N_{Max}}$$

The modified non-uniform mutation is the opposite of the non-uniform mutation in the sense that in this case the probability of mutation increases as the algorithm progresses. This method is more effective against the problem of premature convergence. As the algorithm progresses, diversity tends to decrease and the algorithm focuses on smaller solution areas. The modified non-uniform mutation allows jumps to new, unexplored spaces, avoiding convergence to some local optimum. The equation expresses the calculation of the probability of mutation *p_i_*, increasing with the number of generations i.

$$p_{i}=p_{Min}+\frac{i\left(p_{Max}-p_{Min}\right)}{N_{Max}}$$

For order-based coding, there are mainly four types of mutation, depending on which genes are altered and how they change: swap mutation of any two genes, swap of two adjacent genes, random swap of a set of consecutive genes, or swap of a set of consecutive genes by applying a defined shift to all of them:

- Swap mutation involves exchanging the values of randomly selected genes.
- Adjacent genes swap mutation selects two consecutive genes on the chromosome and reverses their order.
- In inversion mutation, two random positions on the chromosome are chosen and the gene values are swapped between them. Suppose the positions are i and j. The in-terchange operation exchanges the value of gene i with j and vice versa, exchanging genes (i + 1) and (j − 1), (i + 2) with (j − 2) and so on until the interval [i–j] is covered, the last genes exchanged being (i + n) and (j − n), with n = (j − i + 1)/2.
- In shift mutation, a set of consecutive genes to be altered and a direction of displacement (to the right or to the left) is determined. The process consists of each gene in the interval moving to occupy the immediate position until the last gene affected is made to correspond to the first position of the same group of genes. The effect is that the values of all affected genes are shifted one position on the chromosome.

##### Local Search

Local search is a frequently used method in solving combinatorial optimisation problems. Given a solution s_0_, its neighbourhood is explored by searching for a solution s_1_ close to and better than s_0_. The neighbourhood of a solution is formed by the set of solutions that are reached with a small movement or modification in the solution. Solution s_1_ is better than s_0_ if its fitness is closer to the optimum than fitness of the other. The main problem with this technique is the great ease with which the algorithm gets trapped in local optima. There are several possibilities to avoid this outcome:

- Extending the size of the neighbourhood.
- Limiting the number of search iterations.
- Repeating the search algorithm with different starting solutions.

None of them on their own has produced satisfactory results. What generates the best results is to combine the local search technique with other types of mechanisms in what are called hybrid algorithms.

GA is a common choice for global optimization and has been used to search polymer space [158]. It completes a structured search through procedures inspired by the natural evolution of the species. At each iteration, parameter vectors (‘genotypes’) in a population are updated (selection, crossover and mutation) to generate an offspring, followed by an evaluation of the objection function value. Up to date, GA has been used to predict the thermal and optoelectronic properties of neat polymers and fibre-reinforced composites [159,160,161,162,163]. However, despite its huge potential, applications of GA to polymeric nanocomposites are still scarce [133,164,165,166].

Rabothata et al. [167] used GA to optimize both the design parameters and the mechanical properties (elastic modulus and strength) of polymeric nanocomposites. The algorithm was implemented in Matlab and was fairly accurate to find the optimum property values. Recently, Mairpady et al. [168] used an ANN combined with a GA to optimize the concentration of nanofillers and compatibilizing agents of injection moulded high-density polyethylene (HDPE) bionanocomposites filled with nanoTiO_2_ or cellulose nanocrystals. Mechanical properties such as Young’s modulus, tensile strength and fracture strength were optimized with minimum errors and regression values above 95%. This study shows promising results for optimizing the amount and type of nanofillers to be added to polymeric matrices in order to improve mechanical durability.

#### 3.3.3. Gaussian Process

Gaussian process (GP) is a non-parametric stochastic algorithm used for solving non-linear problems [169]. It is one of the Naïve Bayer’s variants, widely used to bridge the gap between computer simulations and physical conditions [170]. GP is characterized by two functions, namely its mean, µ(*x*_i_), and the covariance, c (*x*_i,_ *x*_j_), where i, j vary from 1 to *n*. The parameter *n* is the number of data points and x is the input vector. The GP model can be expressed as:

F(x) = GP (µ(*x*_i_), c (*x*_i,_
*x*_j_))

and the final output can be expressed as

*Y*(*x*) = *F* (*x*) + *err*(*x*)

where *Y*(*x*) is the output of interest and *err*(*x*) is the error related with the dataset noise. The GP does not need setting a hypothesis and finding suitable values for the weights in the framework. It generates a distribution of all the potential functions that are someway consistent with the training data.

Wang et al. [171] investigated the interphase properties of polymer nanocomposites, and used the GP to correlate the feature space with these properties in terms of its viscoelastic and dielectric behaviour. The goal was to minimize the difference between a predicted bulk property and the experimental data. Adaptive optimization was used, which accepts the feedback from the working environment and then works consequently to make improved predictions. The GP was chosen as a surrogate model due to its ability to consider uncertainties and assess the non-linear response with minimum random error.

Hansoge et al. [172] applied the GP to forecast the mechanical behaviour of polymer nanocomposites reinforced with hairy nanoparticles. Polymer–nanoparticle bond strength between, grafting density, chain length and the edge length of the nanoparticles were used as input parameters, whereas the toughness modulus of the resulting composite was the output variable. Training data were derived from molecular dynamics simulations and then the GP regression was performed. The results obtained from the GP and the molecular dynamics simulations were in very good agreement.

Qin et al. [173] studied the effective permittivity of polymeric nanocomposites filled with nanowires within the frequency range of 1–6 GHz. The influence of increasing nanowire concentration on the strain sensitivity and permittivity was analysed using a GP model. GP can also be used for analysing microstructural images of composites in order to extract significant information from them. Gaussian filters have been used for minimizing image and signal noise [174]. Schadler et al. [175] developed a methodology to design polymeric nanocomposites for dielectric applications, and predicted their breakdown strength. FEM and Monte Carlo simulations were performed for the loss functions and the dielectric constant, and the results derived from these simulations were then modelled using GP to study the effect of nanofiller concentration and state of dispersion on the breakdown strength, loss functions and the dielectric constant.

## 4. Conclusions and Future Outlook

The outstanding multi-functional properties of polymeric nanocomposites have made them perfect candidates for a wide number of applications including aerospace, automobile, marine, civil, and many other technologically demanding industries. The increasing request for these nanocomposites demands a comprehensive investigation of their physical, chemical and mechanical behaviour under different environmental conditions. The physical properties, size and shape of the nanofiller and the microstructure of the nanocomposites are important factors that condition their final properties. It requires a lot of time and energy to find high-performance nanocomposites in thousands of combinations, and this process is very hard and long. The ML approach, trained on enormous amounts of data, has been demonstrated to be a very powerful predictive tool for data-driven multi-physical modelling, leading to unique insights and the exploration of their properties beyond the skill of conventional computational and experimental analyses. Recent studies have demonstrated its usefulness for structure–property linkage analysis and for speeding up the design of polymeric nanocomposites. Different ML algorithms including ANN, ANFIS, MLP, CNN, GA, GP, etc., have been successfully applied to create a mapping between the fingerprinted input and the target property. The results demonstrate a very good correlation between the predicted properties and the experimental values, with correlation factors higher than 0.9. Therefore, studies prove that ML algorithms have many advantages versus conventional computing in terms of resolution and cost-effectiveness; they can achieve greater accuracy in the predicted properties, require less expert analysis and fine-tuning and provide superior flexibility since they can be re-trained using a custom dataset for any use case.

Over the past years, most ML-driven approaches were applied to neat polymers and fibre-reinforced composites. Until 2020, less than 100 papers on ML applied to polymer nanocomposites were reported. However, in the past two years, over 200 studies dealing with polymer nanocomposites have been published, many of them related to the prediction of mechanical properties. The prediction accuracy and generalization of ML models are strongly correlated with the quantity and quality of samples in the dataset, and these data are still limited for polymer nanocomposites. Thus, only a few online specific databases such as NanoMine have been built [176]. This problem is expected to be solved in the near future by extracting scientific data untapped in numerous scientific journals using laborious manual excerption or ML-based natural language processing (NLP) techniques [177] or developing advanced simulation methods, such as the multiscale modelling approach [119]. In this regard, a dataset that contains 1254 groups of data on maximum energy storage density of polymer nanocomposites has been very recently established [178]. With growing knowledge on the relationship between microstructures of polymer nanocomposites and their desired properties, other main descriptors, such as the trap state (effects of chemical structures, additives, polymer-nanofiller interface etc.), morphologies (linear, cross-linked, free volume, etc.) and processing conditions should be incorporated into fingerprints to more accurately predict their thermal, electrical, mechanical, tribological properties and so forth. Furthermore, more advanced neural network algorithms (i.e., transfer learning, CNN, etc.) and inverse design methods could be applied for structure–property analysis and property prediction.

Other options are currently been explored. In particular, hybrid machine learning can be applied for the property prediction of polymer nanocomposites. It is based on the idea of combining multiple ML algorithms to increase the overall prediction capability by tuning mutually and generalizing or adapting to unseen data [179]. Ensemble-based methods are an example of hybrid ML, which has already been adopted for the prediction of mechanical response of different types of composites [180,181]. Hybrid machine learning has the potential to surpass individual ML methods in general. Other recent advances in ML comprise adaptive learning. Traditional ML uses training and prediction as two main bases of every algorithm while adaptive learning is founded on reinforcement learning. It spots and learns from the variations in the input and the output values and considers then connected. Adaptive ML gets the feedback from the working environment and then acts consequently to make improved predictions. This has been found to be very promising for solving non-linear, dynamic systems, even in the presence of uncertainties. Multi-scale problems are also very frequent in polymeric nanocomposites since they are made of different phases. Thus, a multiscale analysis method is commonly used to take into account the size effect of the phases or the reinforcement added on the overall behaviour of polymer nanocomposites. Adaptive ML has been successfully used for nanoscale bridging in order to develop efficient nanocomposites [182,183]. Overall, even though the research in this field is still in its infancy, the abovementioned approaches will aid to expand its potential, and a very bright near future is envisaged.

## Figures and Tables

**Table 1:** Representative studies using artificial natural networks (ANNs) to predict the properties of polymeric nanocomposites.

Nanocomposite	ML Model	Input	Output	Ref.
PC/CNT	MLP	*n_PC_*, *n*_CNT_, *w*_CNT_	*ε*	[99]
LLDPE/GNP	ANN	*w*_GNP_; extruder & feeder speed	*K*, T_c_, T_d_, σ_y_	[100]
PEEK/G/Ti	ANN	*w*_G_; *w_Ti_* E_PEEK_, E_G_ SEM images	H, E, σ_y_	[101]
Polymer/SiO_2_	MLP	*w_p_*; *w_SiO2_*; *ω,* t	*η*, *G*	[102]
Epoxy/Al_2_O_3_	ANN	*w_epoxy_ w_Al2O3_*, grain size	H, E, *ε*_m_, σ_y_	[103]
Epoxy/CNT	ANN	E_epoxy_, E_CNT_, ν, Φ_CNT_, l_CNT_	σ	[112]
Polymer/SiO_2_	ANN + ANFIS	*v*_SiO2_; Φ_SiO2_ E_polymer_, E_SiO2_;	*T*	[113]
Epoxy/SiO_2_	ANN	E_epoxy_, E_SiO2_; *v*_epoxy_; *v*_SiO2_; Φ_SiO2_	*T*	[114]
PTFE/CF/TiO_2_	ANN	*v_PTFE_*; *v_T_*_iO2_; t, w_r_	w_L_: µ	[115]
Epoxy/CNT/coir fibre	ANN	*w_epoxy_ w_CNT_*,	*S*	[116]
PLA/GNP	ANN	*w_PLA_*, *w_GNP_* processing parameters	d, H	[117]
Vinyl ester/GNP	ANN	*v_GNP_*; *ω*, *t*	*η*, *G*	[118]
Polymer/SiO_2_	ANN	*v_Polymer_*; *v_T_*_iO2_	E, *ε*_m_, σ_y_	[119]
PVP/SiO_2_	ANN	*w_PVP_*; *w_SiO2_*; *ω*	SA	[120]
PC/G/BC	ANN	*v_PC_*; *v_G_*; *v_BC_*; t, w_r_	w_L_: µ	[121]
Polymer/CNT	ANN	*w_polymer_*; *w_CNT_* Φ_CNT_, l_CNT_; σ_CNT_	σ	[122]
Polymer/nanofiller	CNN	2D images	*η*, *G*; *T_g_*	[123]
Polymer/GNP	ANN	Electric vector	*I*	[124]
PPy/CNT	ANN	*w_PPy_*; *w_CNT_;P*	Flux measurements	[125]
Styrene/AA/CB	ANN	*w_AA_*; *w_CB_*	T_g_, T_c_, T_d_,	[126]
PP/nanoclay	MLP + BP	*w_PP_*; *w_nanoclay_* E_PP_/E_nanoclay_	Mechanical lifetime	[127]
Epoxy/CNT	ANN + BP	*w_CNT_*; extruder & feed speed	R_a_	[128]
Starch/Clay/AgNPs	FF + MLP	*w_starch_; w_Clay_*; *w_AgNO3_*	AgNPs size	[129]
LLDPE/nanoclay	ANN + BP	*w_LLDPE_*; *w_nanoclay_* extruder & feeder speed	E, *ε*_m_, σ_y_	[130]
Polymer/QDs	ANN + MLP	*w_polymer_*; *w_QDs_*	Energy levels Absorption spectrum	[131]
PBA/Bi_2_O_3_	ANN	*w_PBA_*; *w_Bi2O3_*	T_d_, E_F_, σ_F_	[132]
PA-6/Nanoclay	ANN + GA	*w_PA-6_*; *w_nanoclay_* extruder & feeder speed	R_a_	[133]
LLDPE/G/SiO_2_	ANN	*w*_G_; *w_SiO2_*	∆E, δ	[134]
PA-6/Nanoclay	ANFIS	*w_PA-6_; w_nanoclay_* extruder & feeder speed	E	[135]
Polymer/CNT	ANN	*w*_CNT_; *w*_polymer_	E	[136]

*n_m_*: matrix refractive index; *n*_CNT_: CNT refractive index; *w_i_*: weight fraction; *v_i_*: volume fraction; *K*: thermal conductivity; ε: absorption index; E: elastic modulus; T_c_: crystallization temperature; T_d_: decomposition temperature; σ_y_: tensile strength; Φ_CNT_: CNT diameter: l_CNT_: CNT length; ν: Poisson’s ratio; σ: electrical conductivity; *η*: viscosity; *G*: storage modulus; *ω*: frequency; *ε_m_*: maximum strain; *T*: fracture toughness: CF: carbon fibre; w_r_: wear rate; w_L_: wear loss; µ: friction coefficient; *S*: shear modulus; d: density; SA: sound absorption; PVP: polyvinyl pyrrolidone; *I*: current density; PPy: polypyrrol; *P*: pressure; AA: acrylonitrile; CB: carbon black; PP: polypropylene; R_a_: roughness; PBA: Polybenzoxazine; E_F_: flexural modulus; σ_F_: flexural strength; ∆E: band gap; δ: dipole moment.

**Table 2:** Obtaining selection probabilities for a population of six individuals using the roulette technique, with the objective of maximizing the fitness function.

Indiv *i*	F*_i_*	Probability *p_i_*
1	0.5	0.025
2	1	0.075
3	2	0.175
4	3.5	0.35
5	5	0.6
6	8	1.0
∑	20	-

## Data Availability

Not applicable.
